# Registro Multicêntrico Brasileiro de Ablação Septal Alcoólica em Pacientes com Miocardiopatia Hipertrófica Obstrutiva Sintomática – Registro BRASA

**DOI:** 10.36660/abc.20240626

**Published:** 2025-06-06

**Authors:** Pedro Jallad, Marilia Soliani, Pedro Henrique Almeida Marins, Fábio Fernandes, Edmundo Arteaga-Fernandez, Vagner Madrini, Pedro Lemos, Charles Mady, Felix Ramires, Alexandre A. Abizaid, Fábio Sândoli de Brito, Henrique Barbosa Ribeiro

**Affiliations:** 1 Hospital das Clínicas Faculdade de Medicina Universidade de São Paulo São Paulo SP Brasil Instituto do Coração do Hospital das Clínicas da Faculdade de Medicina da Universidade de São Paulo, São Paulo, SP – Brasil; 2 Hospital das Clínicas Faculdade de Medicina Universidade de São Paulo São Paulo SP Brasil Hospital das Clínicas da Faculdade de Medicina da Universidade de São Paulo, (HCFMUSP), São Paulo, SP – Brasil; 3 Hospital Israelita Albert Einstein São Paulo SP Brasil Hospital Israelita Albert Einstein, São Paulo, SP – Brasil; 4 Faculdade de Medicina Universidade de São Paulo São Paulo SP Brasil Faculdade de Medicina da Universidade de São Paulo, São Paulo, SP – Brasil; 5 Hospital Sírio-Libanês São Paulo SP Brasil Hospital Sírio-Libanês, São Paulo, SP – Brasil

**Keywords:** Intervenção Coronária Percutânea, Ablação por Cateter, Cardiomiopatia Hipertrófica

## Abstract

**Fundamento:**

A ablação septal alcoólica (ASA) é uma alternativa à miectomia cirúrgica para pacientes com cardiomiopatia hipertrófica obstrutiva (CMHO) sintomática e obstrução significativa da via de saída do ventrículo esquerdo (VSVE). Embora amplamente estudada em outros países, ainda há poucos dados sobre os resultados da ASA no Brasil.

**Objetivo:**

Avaliar a segurança e a eficácia da ASA em pacientes sintomáticos com CMHO, tratados com terapia medicamentosa otimizada, utilizando técnicas atuais em diferentes centros brasileiros.

**Métodos:**

Foram incluídos pacientes com CMHO e angina (classificação da *Canadian Cardiovascular Society* [CCS]) ou dispneia (classificação da *New York Heart Association* [NYHA]) em classe funcional acima de II, sem resposta ao tratamento medicamentoso otimizado. O desfecho primário de eficácia foi definido como a redução superior a 50% no gradiente máximo da VSVE em repouso, com valor final <50 mmHg. Os pacientes foram classificados como responsivos ou não responsivos. Um valor de p<0.05 foi considerado estatisticamente significativo.

**Resultados:**

Um total de 41 pacientes (idade mediana de 66,4 anos; 73% mulheres) foi submetido à ASA. No início, 93,2% estavam em classe funcional III/IV da NYHA ou CCS. A fração de ejeção do ventrículo esquerdo (FEVE) média era de 66,4%, e o gradiente médio da VSVE era de 88,4 mmHg. Após 12 meses, 92,8% apresentaram melhora para classe funcional I/II da NYHA ou CCS (p<0,01). O gradiente médio da VSVE caiu de 88,4 mmHg para 27,0 mmHg (p=0,003), e a espessura do septo interventricular (SIV) diminuiu de 19,3 mm para 14,7 mm (p=0,048). Pacientes responsivos apresentaram gradientes basais menores (73,4 vs 112,6 mmHg, p=0,04) e menos hospitalizações (21,1% vs 82,4%, p=0,04). Bloqueio atrioventricular completo ocorreu em 16,7% dos casos, e 4,8% necessitaram de marcapasso. Não houve óbitos durante o seguimento mediano de 394 dias. Na última avaliação presencial, 78,4% estavam em classe funcional I/II.

**Conclusões:**

A ASA é uma opção segura e eficaz para alívio dos sintomas em pacientes selecionados com CMHO. O procedimento reduz o gradiente da VSVE e a espessura septal. Pacientes com gradientes basais mais elevados apresentaram menor taxa de resposta.

## Graphical abstract

**Figura Central:**
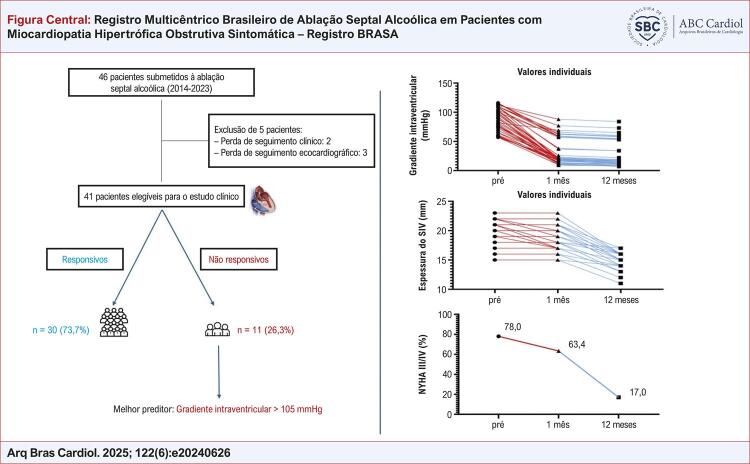
Registro Multicêntrico Brasileiro de Ablação Septal Alcoólica em Pacientes com Miocardiopatia Hipertrófica Obstrutiva Sintomática – Registro BRASA

## Introdução

Em pacientes com cardiomiopatia hipertrófica obstrutiva (CMHO), a obstrução dinâmica da via de saída do ventrículo esquerdo (VSVE) tem papel central na origem dos sintomas, como angina, síncope e dispneia. O tratamento farmacológico é amplamente utilizado e inclui betabloqueadores, bloqueadores dos canais de cálcio e, mais recentemente, mavacamten.^1-3^ No entanto, cerca de 10% dos pacientes não respondem à terapia medicamentosa.^4^ Para esses casos, foram desenvolvidas terapias de redução septal (p.ex., a miectomia cirúrgica e a ablação septal alcoólica [ASA]) com o objetivo de aliviar os sintomas e melhorar os desfechos clínicos.^5,6^

A miectomia cirúrgica ainda é considerada o tratamento padrão-ouro para a cardiomiopatia hipertrófica obstrutiva, especialmente em pacientes mais jovens com gradientes basais elevados, hipertrofia septal acentuada ou outras indicações cirúrgicas associadas.^7^ No entanto, os avanços na técnica de ASA (p.ex., o uso de ecocardiografia com contraste e técnicas mais precisas de infusão de álcool) melhoraram os resultados do procedimento. Em pacientes selecionados, a ASA demonstrou taxas de mortalidade a longo prazo semelhantes (em torno de 1%) e menor incidência de complicações periprocedimento.^5,6^ A miectomia costuma ser preferida em pacientes jovens, com baixo risco cirúrgico e espessura septal superior a 25 mm. Já a ASA é frequentemente indicada para pacientes mais idosos, de alto risco, com anatomia adequada, por ser um procedimento menos invasivo.^5^

As diretrizes atuais reconhecem tanto a miectomia cirúrgica quanto a ASA como opções terapêuticas válidas, mas a avaliação cuidadosa por uma equipe especializada é essencial. Essa análise deve levar em conta o estado geral do paciente, os riscos específicos de cada procedimento e a presença de alterações na valva mitral ou outras particularidades anatômicas.^5^ Apesar da experiência acumulada mundialmente, ainda faltam dados atualizados sobre o uso da ASA no tratamento da CMHO no Brasil.

O objetivo deste estudo é avaliar a segurança e a eficácia da ASA em uma série de pacientes sintomáticos com CMHO, em uso de terapia medicamentosa otimizada, utilizando técnicas atuais em diferentes centros brasileiros.

## Métodos

### População do estudo

Foram incluídos, de forma retrospectiva, os pacientes submetidos à ASA entre janeiro de 2014 e dezembro de 2023, registrados em um banco de dados específico. Os procedimentos foram realizados em quatro centros terciários de referência no Brasil: Instituto do Coração do Hospital das Clínicas da Faculdade de Medicina da Universidade de São Paulo (InCor-HCFMUSP), Hospital Sírio-Libanês, Hospital São Camilo e Hospital Samaritano Paulista. O estudo foi aprovado pelo comitê de ética local de cada centro participante.

Foram considerados elegíveis os pacientes com 18 anos ou mais e diagnóstico de CMHO confirmado por ecocardiografia bidimensional e/ou ressonância magnética cardíaca (RMC). O diagnóstico exigia espessura do septo interventricular (SIV) ≥15 mm ou ≥13 mm em pacientes com parente de primeiro grau adulto com diagnóstico de CMHO, na ausência de outras doenças cardíacas ou sistêmicas associadas à hipertrofia ventricular esquerda secundária.

A obstrução foi definida como gradiente de pressão na VSVE ≥50 mmHg, em repouso ou após provocação. Os pacientes elegíveis também deveriam estar sintomáticos e refratários à terapia medicamentosa otimizada, incluindo pelo menos um agente cronotrópico negativo na dose máxima tolerada, e apresentar dispneia em classe funcional >II da NYHA ou angina em classe >II da CCS.^6^ Durante o período do estudo, o mavacamten ainda não estava disponível para os pacientes dessa coorte.

Além disso, os pacientes deveriam ser considerados aptos para terapia de redução septal com base em indicação clínica e anatomia coronariana favorável. Isso incluía a presença de um ramo septal acessível — geralmente originado da artéria descendente anterior (ADA) — e ausência de alterações significativas na valva mitral ou de outras condições que exigissem cirurgia cardíaca.^7,8^

Os critérios de exclusão incluíram ausência de dados de seguimento após a internação inicial (como falta de acompanhamento clínico ou ecocardiográfico) e anatomia coronariana desfavorável para a ASA, como ausência de ramo septal adequado, espessura septal >30 mm ou necessidade de procedimentos cirúrgicos concomitantes.

### Procedimento de ASA

Todos os procedimentos seguiram a técnica padrão de ASA, realizados com auxílio de ecocardiografia com contraste, conforme previamente descrito.^9^ Os pacientes receberam sedação ou anestesia geral, com orientação por ecocardiografia transesofágica ou transtorácica com contraste. Após obtenção dos acessos arterial e venoso, iniciou-se anticoagulação sistêmica com heparina não fracionada na dose de 100 UI/kg.

Foi introduzido um eletrodo de marcapasso temporário (5F ou 6F) no ventrículo direito. Um cateter *pigtail* (5F ou 6F) foi posicionado no ventrículo esquerdo por um acesso secundário, e um introdutor guia esquerdo de 6F (*Judkins Left* [JL] ou *Extra-Backup* [EBU]) foi inserido, por acesso primário, na aorta ascendente. O gradiente de pressão na VSVE foi monitorado continuamente durante todo o procedimento.

Foi realizada angiografia inicial para identificar a origem do ramo septal perfurante. Em seguida, um fio-guia de 0,014” foi avançado até o ramo septal-alvo. Um balão *over-the-wire* (1,5-2,5 mm × 8-12 mm) foi então insuflado dentro do ramo selecionado. A perfusão miocárdica da área-alvo foi avaliada por ecocardiografia com contraste. Em 58,5% dos casos, utilizou-se SonoVue (Bracco); nos demais, foi injetada uma mistura de soro fisiológico com contraste através do eixo do cateter-balão, com ecocardiografia em tempo real.

Se o balão estivesse adequadamente posicionado, de 1 a 3 ml de etanol eram injetados lentamente à taxa de 1 ml por minuto. Dez minutos após a injeção, o balão era desinsuflado, e nova angiografia coronária era realizada para avaliar o resultado. O procedimento era considerado bem-sucedido quando havia redução de pelo menos 50% no gradiente de pressão da VSVE em relação ao valor basal. Caso o resultado fosse insatisfatório, o procedimento poderia ser repetido em outro ramo septal.

Todos os pacientes foram monitorados em unidade de terapia intensiva ou unidade coronariana por um período de 24 a 48 horas após o procedimento. Os níveis plasmáticos de creatinofosfoquinase (CPK) total e de creatinofosfoquinase fração MB (CK-MB) foram dosados antes do procedimento e a cada 6 horas nas 36 horas seguintes.

As avaliações ecocardiográficas foram realizadas antes da ASA e repetidas 1 mês e 1 ano após o procedimento. Todas as medições seguiram as recomendações propostas pela *American Society of Echocardiography.*^10-12^

### Definições e desfechos do estudo

O desfecho primário de eficácia foi definido como uma redução >50% no gradiente máximo da VSVE em repouso, com gradiente final <50 mmHg. Esse critério foi utilizado para determinar o sucesso do procedimento e foi avaliado aos 1 e 12 meses após a ASA. Com base nesse parâmetro, os pacientes foram classificados como responsivos ou não responsivos.

A melhora dos sintomas cardíacos foi avaliada pela redução de, no mínimo, uma classe funcional nas classificações da NYHA ou da CCS após o procedimento.

O desfecho primário de segurança foi a ocorrência de complicações, incluindo bloqueio atrioventricular completo, fibrilação ventricular, implante de marcapasso definitivo ou desfibrilador cardioversor, tamponamento cardíaco, cirurgia cardíaca de emergência ou morte cardiovascular.

### Análise estatística

As análises estatísticas foram realizadas utilizando o GraphPad versão 10.2.2 e o Microsoft Excel 2010 (Microsoft Corporation, Redmond, Washington). As variáveis categóricas são apresentadas em frequências absolutas e relativas.

A normalidade foi avaliada pelo teste de Shapiro-Wilk. Os dados com distribuição normal são apresentados como média±desvio padrão, enquanto os dados sem distribuição normal são expressos como mediana e intervalo interquartil (IIQ).

Para comparar variáveis contínuas entre dois grupos independentes, utilizou-se o teste t de Student não pareado nos casos de distribuição normal e o teste de Mann-Whitney para distribuições não normais. Para comparações envolvendo mais de dois momentos no tempo, foi utilizada a análise de variância (ANOVA) para medidas repetidas quando os dados apresentavam distribuição normal.

Variáveis categóricas em amostras independentes foram comparadas por meio do teste do qui-quadrado. Para dados categóricos dependentes, utilizou-se o teste Q de Cochran.

As curvas de sobrevida de Kaplan-Meier foram analisadas com o teste de log-rank. Em todas as análises, valores de p<0,05 foram considerados estatisticamente significativos.

## Resultados

### Características basais e procedimentos de ASA

**Figura 1:**
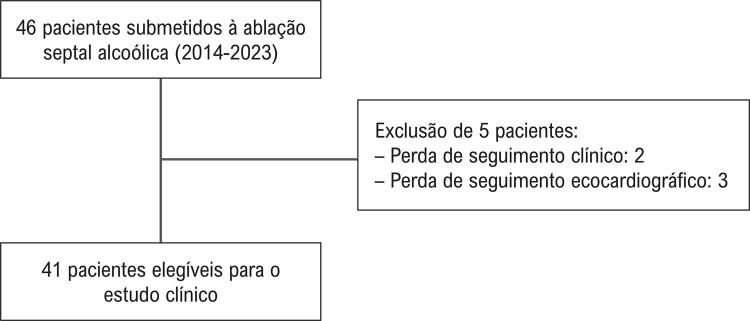
– Fluxograma da população estudada.

Um total de 46 pacientes foi incluído no registro. No entanto, cinco foram excluídos por ausência de dados de seguimento — dois sem informações clínicas e três sem dados ecocardiográficos (Figura 1). A média de idade foi de 66 anos, e a maioria dos pacientes era do sexo feminino (73%). A principal indicação para a ASA foi dispneia aos esforços. A maioria dos pacientes encontrava-se em classe funcional III/IV da NYHA (78,1%) ou da CCS (51,1%). Além disso, 24,3% tinham história de síncope não relacionada a arritmias. No total, 93,2% apresentavam sintomas avançados.

As comorbidades mais frequentes foram hipertensão arterial, diabetes mellitus, fibrilação atrial e doença arterial coronariana. Alguns pacientes haviam sido submetidos previamente à angioplastia coronária ou apresentavam distúrbios de condução, como bloqueio de ramo esquerdo. Um número reduzido utilizava cardioversores desfibriladores implantáveis (CDI). Todos os pacientes faziam uso de betabloqueadores ou bloqueadores dos canais de cálcio na linha de base, sendo os betabloqueadores mais utilizados. Uma proporção menor necessitava de amiodarona ou anticoagulantes orais. A RMC foi utilizada para avaliar a fibrose miocárdica. A área média de fibrose foi de 11,7%, sem diferenças significativas entre os grupos (Tabela 1).

**Tabela 1:** – Características clínicas e ecocardiográficas/RMC na linha de base

Variável	Total (n=41)	Responsivos (n=30)	Não responsivos (n=11)	Valor p
Idade (anos)	66,4±5,9	64,7±6,8	65,4±7,2	0,64
Sexo feminino, n (%)	30 (73,1)	24 (80,0)	6 (54,5)	0,81
Hipertensão arterial, n (%)	27 (65,8)	21 (70,0)	6 (54,5)	0,74
Doença arterial coronariana, n (%)	7 (17,0)	3 (10,0)	4 (36,3)	0,67
ICP prévia, n (%)	3 (7,2)	2 (6,6)	1 (9,0)	0,36
Diabetes melito, n (%)	9 (21,0)	7 (23,3)	2 (18,1)	0,68
Dispneia classe NYHA III/IV, n (%)	32 (78,0)	25 (83,3)	7 (63,6)	0,68
Angina classe CCS III/IV, n (%)	21 (51,2)	15 (50,0)	6 (54,5)	0,75
Fibrilação atrial, n (%)	4 (9,7)	3 (10,0)	1 (9,0)	0,75
Bloqueio de ramo, n (%)	6 (14,6)	4 (13,3)	2 (18,1)	0,45
Gradiente da VSVE em repouso (mmHg)	88,4±23,0	73,4±23,4	112,6±40,2	0,04
FEVE (%)	66,4±11,1	65,3±9,1	68,2±10,4	0,92
Espessura do SIV (mm)	19,3±3,2	15,3±2,2	16,5±3,9	0,56
Betabloqueador, n (%)	35 (85,3)	25 (83,3)	10 (90,9)	0,33
Bloqueador de canal de cálcio, n (%)	10 (24,3)	8 (26,6)	2 (18,1)	0,41
Amiodarona, n (%)	6 (14,6)	4 (13,3)	2 (18,1)	0,55
Anticoagulante oral, n (%)	3 (7,3)	3 (13,3)	1 (9,0)	0,62
Fibrose por RMC (área total, %)	11,7±4,2	10,4±3,7	12,1±2,2	0,48
Fibrose por RMC (gramas)	13,9±3,8	13,4±4,1	15,8±4,1	0,37

Os dados são expressos como média (DP) ou como número absoluto e percentual, conforme apropriado. CCS: Canadian Cardiovascular Society; CDI: cardioversor desfibrilador implantável; DP: desvio padrão; FEVE: fração de ejeção do ventrículo esquerdo; ICP: intervenção coronária percutânea; NYHA: New York Heart Association; RMC: ressonância magnética cardíaca; SIV: septo interventricular; VSVE: via de saída do ventrículo esquerdo.

A ecocardiografia Doppler basal confirmou a presença de CMHO grave, caracterizada por gradiente elevado de pressão na VSVE e aumento da espessura do SIV. A maioria dos pacientes (92,6%) apresentava fração de ejeção do ventrículo esquerdo (FEVE) preservada, e o átrio esquerdo encontrava-se moderadamente dilatado (Tabela 1). A mediana de tempo de internação foi de 6 dias, variando de 3 a 15 dias. Os exames laboratoriais mostraram valores estáveis de hemoglobina, hematócrito e creatinina durante todo o período de internação. Refratariedade ao tratamento farmacológico foi observada em 18,2% dos pacientes. Esses indivíduos estavam em uso combinado de betabloqueadores e bloqueadores dos canais de cálcio, mas continuavam a apresentar gradientes elevados na VSVE, mesmo com manejo medicamentoso otimizado (Tabela 1).

O procedimento de ASA foi realizado com um volume mediano de etanol de 1,9 cc por paciente. A maioria dos pacientes (n=36) foi submetida à ASA de um único ramo septal, enquanto cinco necessitaram do tratamento de dois segmentos septais no mesmo procedimento. Os níveis pós-procedimento de pico de CK-MB e troponina estavam elevados, mas permaneceram dentro da faixa esperada (Tabela 2). A artéria radial foi utilizada como via de acesso preferencial em 73,1% dos casos, e a maior parte dos procedimentos foi realizada com cateter 6F (Tabela 2).

**Tabela 2:** – Características do procedimento de ablação septal alcoólica

Características do procedimento	Total (n=41)	Responsivos (n=30)	Não responsivos (n=11)	Valor p
Número médio de ramos septais tratados por paciente	1,12±0,07	1,13±0,09	1,09±0,04	0,83
Um ramo septal tratado, n (%)	36 (87,8)	26 (86,6)	10 (90,9)	
Dois ramos septais tratados, n (%)	5 (12,2)	4 (13,4)	1 (9,1)	
Volume de etanol por paciente (ml), mediana [IIQ]	1,9 [1,6-2,4]	1,8 [1,6-2,3]	2,0 [1,8-2,4]	0,54
Pico de CK-MB (ng/ml), mediana [IIQ]	157 [126-254]	152 [113-286]	169 [129-304]	0,42
Pico de troponina (ng/l), mediana [IIQ]	10,860 [9,576-12,239]	10,539 [9,284-12,482]	11,285 [9,896-12,129]	0,39
Acesso arterial primário, n (%)				0,71
Radial	30 (73,1)	23 (76,6)	7 (63,6)	
Femoral	11 (26,8)	7 (23,4)	4 (36,4)	
Diâmetro do cateter, n (%)				0,63
6F	38 (92,7)	28 (93,3)	10 (90,9)	
5F	3 (7,3)	2 (6,7)	1 (9,1)	

Os dados são apresentados como número absoluto e percentual, média (DP) ou mediana [IIQ], conforme apropriado. CK-MB: creatinofosfoquinase fração MB; DP: desvio padrão; IIQ: intervalo interquartil.

### Desfechos de eficácia

**Figura 2:**
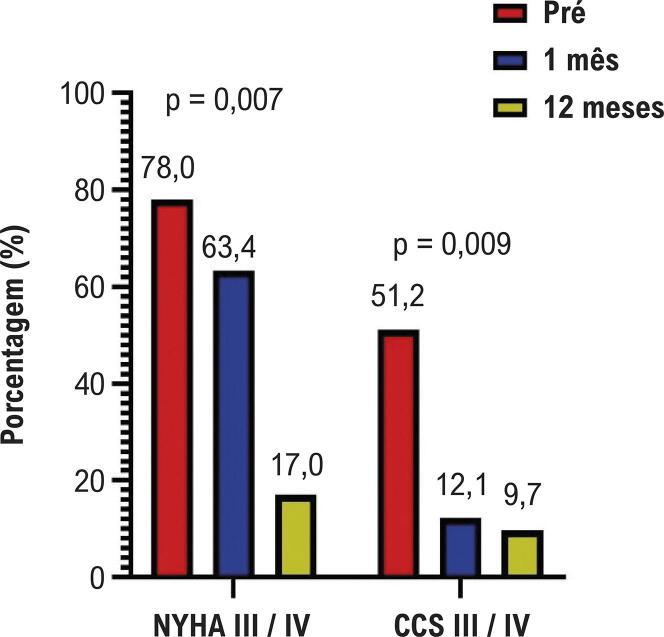
– Seguimento clínico. CCS: Cardiovascular Canadian Society; NYHA: New York Heart Association; Pré: antes do procedimento.

**Figura 3:**
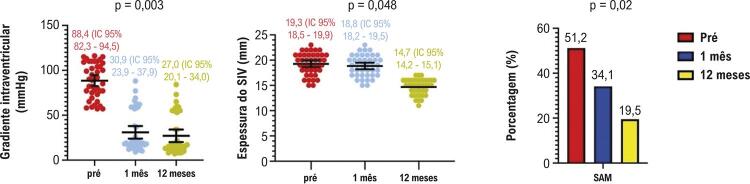
– Alterações ecocardiográficas ao longo do tempo. CCS: Cardiovascular Canadian Society; NYHA: New York Heart Association; SAM: movimento sistólico anterior; SIV: septo interventricular.

Após o procedimento de ASA, observou-se uma redução significativa no número de pacientes classificados como classe funcional III/IV da NYHA. Em 1 mês, 26,8% ainda se encontravam nessas classes avançadas, proporção que caiu para 17,0% aos 12 meses. Foi observada melhora semelhante na classificação da CCS, com 12,1% e 9,7% dos pacientes permanecendo em classe III/IV aos 1 e 12 meses, respectivamente (Figura 2). As avaliações ecocardiográficas mostraram redução expressiva no gradiente de pressão da VSVE, que passou de 88,4 mmHg para 27,0 mmHg. A espessura do SIV também apresentou queda, de 19,3 mm para 14,7 mm aos 12 meses após o procedimento (Figura 3).

O movimento sistólico anterior (SAM) da valva mitral, que reflete o efeito Venturi causado pela obstrução da VSVE, também apresentou redução significativa — de 51,2% antes do procedimento para 19,5% aos 12 meses. Vale destacar que a incidência de SAM continuou a diminuir entre o primeiro e o décimo segundo mês após a ASA, caindo de 34,1% para 19,5% (Figura 3).

**Figura 4:**
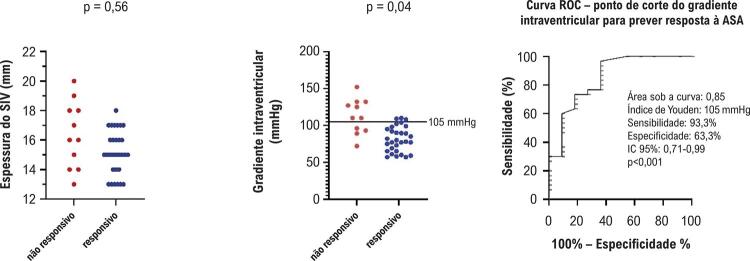
– Comparação entre pacientes responsivos e não responsivos (parâmetros ecocardiográficos na linha de base).

**Figura 5:**
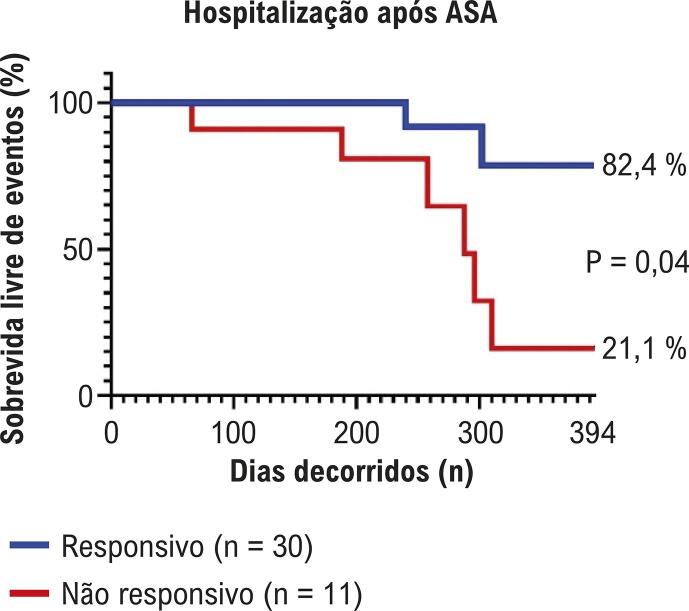
– Comparação entre hospitalização de responsivos e não responsivos após procedimento ablação septal alcoólica (ASA).v

Com base na redução de pelo menos 50% no gradiente de pressão da VSVE e em um valor absoluto final <50 mmHg, os pacientes foram classificados em dois grupos: responsivos (30 pacientes, 73,0%) e não responsivos (11 pacientes, 27,0%). Pacientes responsivos apresentaram gradiente basal da VSVE menor em comparação aos não responsivos (73,4±23,4 mmHg vs 112,6±40,2 mmHg) (Tabela 1 e Figura 4). No entanto, não houve diferença significativa na espessura basal do SIV entre os grupos, conforme medição ecocardiográfica (15,3±2,2 mm vs 16,5±3,9 mm) (Tabela 1 e Figura 4). A análise da curva ROC (*receiver operating characteristic*) identificou um gradiente basal de 105 mmHg como ponto de corte ideal para prever a resposta à ASA (Figura 4). Durante o seguimento mediano de 394 dias, a análise de Kaplan-Meier mostrou que pacientes não responsivos apresentaram taxas significativamente maiores de hospitalização em comparação aos responsivos (Figura 5).

Dois pacientes não responsivos foram submetidos à miectomia cirúrgica após a ASA. Ambos apresentavam gradientes residuais na VSVE >50 mmHg. Após a cirurgia, o gradiente não foi mais identificado nas avaliações ecocardiográficas, e nenhum evento adverso foi registrado durante o procedimento ou no seguimento.

### Desfechos de segurança

Bloqueio atrioventricular completo ocorreu no período periprocedimento em sete pacientes (17%). Dentre esses, dois (4,8%) retornaram ao ritmo sinusal em até 24 horas, e três (7,3%) em até 48 horas. O implante de marcapasso definitivo foi necessário em dois pacientes (4,8%). Durante o estudo, foi adotado um período de observação de cinco dias antes da decisão pelo implante do marca-passo. Fibrilação ventricular ocorreu durante o procedimento em quatro pacientes (9,7%), mas foi revertida com sucesso em todos os casos, com boa recuperação pós-operatória. Não houve casos de tamponamento cardíaco, cirurgia cardíaca de emergência ou óbitos durante a internação. Também não foram registrados óbitos durante o seguimento mediano de 394 dias (Tabela 3).

**Tabela 3:** – Desfechos de segurança do procedimento de ablação septal alcoólica

Desfechos de segurança	Total (n=41)	Responsivos (n=30)	Não responsivos (n=11)
Bloqueio atrioventricular completo, n (%)	7 (17)	5 (16,6)	2 (18,1)
Implante de novo marcapasso, n (%)	2 (4,8)	1 (3,3)	1 (9)
Fibrilação ventricular, n (%)	4 (9,7)	3 (10)	1 (9)
Tamponamento cardíaco	—	—	—
Cirurgia cardíaca de urgência	—	—	—
Mortalidade	—	—	—

Os dados são apresentados como número absoluto e percentual.

## Discussão

Os principais achados deste estudo foram: i) houve melhora clínica significativa após o procedimento de ASA, com menos de 20% dos pacientes permanecendo em classe funcional III/IV da CCS ou da NYHA aos 12 meses; ii) a avaliação ecocardiográfica demonstrou redução evidente do gradiente de pressão da VSVE, acompanhada de diminuição da espessura do SIV e redução marcada do SAM da valva mitral; iii) 73% dos pacientes apresentaram resposta positiva à ASA, o que se associou a menor número de hospitalizações no seguimento, sendo o gradiente intraventricular basal <105 mmHg o melhor preditor de resposta; iv) a ASA mostrou-se um procedimento seguro nesta coorte, com baixa taxa de complicações e ausência de mortalidade.

A CMHO é uma condição heterogênea e com alta prevalência em nossa população. Embora a maioria dos pacientes permaneça estável com tratamento medicamentoso, cerca de 10% eventualmente requerem terapias de redução septal, como miectomia cirúrgica ou ASA. Nas últimas décadas, a técnica de ASA evoluiu e incorporou melhorias, como o uso da ecocardiografia com contraste e volumes menores de injeção de álcool. No entanto, ainda há escassez de dados avaliando pacientes consecutivos tratados com ASA em nossa região.^11-13^ Nossa coorte, embora limitada a 41 pacientes, incluiu indivíduos com CMHO em estágio avançado, caracterizada por elevada carga de sintomas — 78% estavam em classe funcional III/IV da NYHA e 51% em classe III/IV da CCS — e extensão média de fibrose de 11,7%. O tamanho reduzido da amostra provavelmente reflete uma baixa taxa de encaminhamento para terapias de redução septal, seja por miectomia cirúrgica ou ASA, em pacientes com CMHO.

Durante a ASA, a redução aguda do gradiente da VSVE provavelmente ocorre devido ao atordoamento ou infarto septal^2-5^ e a alterações na dinâmica de ejeção do ventrículo esquerdo.^14^ Esses fatores podem contribuir para o alívio dos sintomas no curto prazo. Conforme descrito na literatura, o afinamento progressivo do septo e o remodelamento do ventrículo esquerdo são os principais responsáveis pela redução sustentada do gradiente observada aos 12 meses.^14^ Essa melhora contínua é reforçada pela redução persistente dos sintomas e pela queda progressiva do gradiente da VSVE ao longo do tempo.^15^

Não foi encontrada associação significativa entre a espessura do septo e o sucesso do procedimento. Esse achado contrasta com estudos anteriores que sugerem que espessura do SIV >30 mm pode reduzir a eficácia da ASA, tornando a miectomia a abordagem preferencial nesses casos. No entanto, os resultados cirúrgicos em pacientes com septos muito espessados também costumam ser subótimos.^13,16^ Vale destacar que nosso estudo não incluiu pacientes com espessura de SIV >30 mm. Isso pode refletir um viés de seleção, já que esses pacientes provavelmente foram encaminhados diretamente para tratamento cirúrgico.

O procedimento de ASA foi bem-sucedido — definido como redução superior a 50% no gradiente da VSVE e valor absoluto final <50 mmHg — em 73% dos pacientes da nossa coorte (n=30), resultado compatível com estudos anteriores.^17-19^ Conforme também relatado na literatura,^2,20^ os pacientes que responderam à ASA apresentaram melhora clínica, com cerca de 50% alcançando classe funcional I da NYHA.^21,22^ A maioria dos pacientes responsivos apresentava gradientes basais de VSVE <100 mmHg antes do procedimento.

Dados recentes de uma coorte com 1.346 pacientes submetidos à ASA, acompanhados por uma média de 5,8±4,7 anos, mostraram que indivíduos com gradiente basal de VSVE ≥100 mmHg apresentaram maiores gradientes residuais e maior probabilidade de necessitar de nova ASA ou miectomia complementar, em comparação com aqueles com gradientes <100 mmHg.^14^ No nosso estudo, foi observada uma correlação negativa entre o gradiente basal da VSVE e os desfechos da ASA. Identificamos um possível ponto de corte de 105 mmHg como preditor de sucesso do procedimento. Esse achado reforça a importância da avaliação do gradiente basal da VSVE na seleção de candidatos à ASA, embora ainda seja necessária confirmação em estudos com maior número de pacientes.

No presente estudo, a ASA foi realizada com técnicas contemporâneas por equipes experientes em centros terciários.^11,16,23,24^ O uso da ecocardiografia transtorácica associada à injeção de contraste aumentou a precisão do procedimento. Além disso, a utilização de balões intracoronarianos para a injeção seletiva de álcool no ramo septal-alvo contribuiu para maior segurança. Esses avanços técnicos colaboraram para a baixa taxa de complicações observada em nossa coorte. O implante de marca-passo definitivo foi necessário em apenas 4,8% dos pacientes, representando uma redução significativa em comparação com taxas relatadas em estudos anteriores. Marca-passos temporários foram utilizados em 17% dos casos, o que reforça a importância do monitoramento intensivo nas primeiras 48 horas para avaliar a necessidade de implante definitivo. A recuperação do ritmo sinusal nesse período de observação pode depender de diversos fatores, como a extensão do dano miocárdico, as características do paciente e a técnica utilizada.^24^

Este estudo apresenta algumas limitações. Primeiramente, trata-se de uma análise retrospectiva com uma amostra pequena de 41 pacientes submetidos a um único procedimento, o que pode ter introduzido viés de seleção. Em segundo lugar, a ausência de testes de esforço após o procedimento representa uma limitação na avaliação da melhora dos sintomas. Não foi utilizado um questionário padronizado; os dados sobre sintomas basearam-se nas classificações da NYHA e da CCS, a partir dos relatos subjetivos dos pacientes durante consultas clínicas de rotina. Além disso, a síncope como indicação primária para realização da ASA não foi registrada em nosso banco de dados, impossibilitando a avaliação de sua prevalência nesta coorte. Por fim, houve perda de dados clínicos e ecocardiográficos após 12 meses, o que limitou a análise dos desfechos em longo prazo.

## Conclusão

Quando realizada com indicações adequadas e técnicas avançadas (p.ex., a ecocardiografia com contraste), a ASA é um procedimento seguro e eficaz, com baixa taxa de complicações. Proporciona melhora significativa nos parâmetros ecocardiográficos, incluindo a redução do gradiente da VSVE e da espessura do SIV, resultando em alívio dos sintomas. Este estudo identificou um gradiente basal da VSVE <105 mmHg como possível preditor de desfechos favoráveis com a ASA. Esses achados reforçam a importância da seleção criteriosa dos pacientes e da utilização de técnicas contemporâneas para otimizar os resultados clínicos.

### Perspectivas clínicas

O registro BRASA destaca o papel crescente da ASA como tratamento seguro e eficaz para pacientes com CMHO sintomática que não respondem à terapia medicamentosa. O estudo demonstra que a ASA promove alívio dos sintomas e melhora dos parâmetros ecocardiográficos, incluindo a redução do gradiente da VSVE e da espessura do SIV, reforçando seu potencial para melhorar a qualidade de vida em pacientes selecionados.

Um gradiente basal da VSVE <105 mmHg foi identificado como possível preditor de desfechos favoráveis, reforçando sua utilidade na seleção de pacientes. O estudo também destaca o valor das técnicas contemporâneas de ASA — como a ecocardiografia com contraste e a infusão precisa do álcool — que contribuem para maior segurança e eficácia do procedimento. As baixas taxas de complicações, especialmente a menor necessidade de implante de marcapasso, ressaltam a importância da padronização técnica e da experiência das equipes envolvidas. Novos estudos são necessários para confirmar esses achados, definir melhor o papel da ASA em comparação a terapias emergentes, como o mavacamten, e esclarecer sua posição na linha de cuidado da CMHO.
