# Supplementary material for: Registro Multicêntrico Brasileiro de Ablação Septal Alcoólica em Pacientes com Miocardiopatia Hipertrófica Obstrutiva Sintomática – Registro BRASA
Source: Arq Bras Cardiol. 2025 Jun 6;122(6):e20240626. [Article in Portuguese] doi: 10.36660/abc.20240626 (PMC12212378; doi:10.36660/abc.20240626)
Supplement: Translation

# Translation: Brazilian Multicenter Registry of Alcohol Septal Ablation for Patients with Symptomatic Hypertrophic Obstructive Cardiomyopathy – BRASA Registry

**DOI:** 10.36660/abc.20240626i

**Authors:** Pedro Jallad, Marilia Soliani, Pedro Henrique Almeida Marins, Fábio Fernandes, Edmundo Arteaga-Fernandez, Vagner Madrini, Pedro Lemos, Charles Mady, Felix Ramires, Alexandre A. Abizaid, Fábio Sândoli de Brito, Henrique Barbosa Ribeiro

**Affiliations:** 1 Brazil Instituto do Coração do Hospital das Clínicas da Faculdade de Medicina da Universidade de São Paulo, São Paulo, SP – Brazil; 2 Brazil Hospital das Clínicas da Faculdade de Medicina da Universidade de São Paulo (HCFMUSP), São Paulo, SP – Brazil; 3 Brazil Hospital Israelita Albert Einstein, São Paulo, SP – Brazil; 4 Brazil Faculdade de Medicina da Universidade de São Paulo, São Paulo, SP – Brazil; 5 Brazil Hospital Sírio-Libanês, São Paulo, SP – Brazil

**Keywords:** Intervenção Coronária Percutânea, Catheter Ablation, Hypertrophic Cardiomyopathy

## Abstract

**Background:**

Alcohol septal ablation (ASA) is an alternative to surgical myectomy for patients with symptomatic hypertrophic obstructive cardiomyopathy (HOCM) and significant left ventricular (LV) outflow tract (LVOT) obstruction. Although widely studied worldwide, data on ASA outcomes in Brazil are still limited.

**Objective:**

To assess the safety and effectiveness of ASA in symptomatic HOCM patients receiving optimal medical therapy using current techniques across multiple centers in Brazil.

**Methods:**

Patients with HOCM and angina (classified by the Canadian Cardiovascular Society [CCS]) or dyspnea (classified by the New York Heart Association [NYHA]) class >II who did not respond to optimal medical treatment were included. The primary efficacy endpoint was defined as a reduction of more than 50% in the maximum resting LVOT gradient, with a final gradient <50 mmHg. Patients were classified as responders or nonresponders. A p-value <0.05 was considered statistically significant.

**Results:**

A total of 41 patients (median age 66.4 years; 73% female) underwent ASA. At baseline, 93.2% were in NYHA class III/IV or CCS class III/IV. The mean LV ejection fraction (LVEF) was 66.4%, and the mean LVOT gradient was 88.4 mmHg. After 12 months, 92.8% had improved to NYHA class I/II or CCS class I/II (p<0.01). The mean LVOT gradient dropped from 88.4 mmHg to 27.0 mmHg (p=0.003), and interventricular septum (IVS) thickness decreased from 19.3 mm to 14.7 mm (p=0.048). Responders had lower baseline gradients (73.4 vs 112.6 mmHg, p=0.04) and fewer hospitalizations (21.1% vs 82.4%, p=0.04). Complete atrioventricular block occurred in 16.7% of patients, and 4.8% required pacemakers. No deaths were reported during a median follow-up of 394 days. At the last in-person evaluation, 78.4% were in functional class I/II.

**Conclusions:**

ASA is a safe, effective option for relieving symptoms in selected HOCM patients. It reduces LVOT gradient and septal thickness. Patients with higher baseline gradients were less likely to respond to the procedure.

## Introduction

In patients with hypertrophic obstructive cardiomyopathy (HOCM), dynamic obstruction of the left ventricular (LV) outflow tract (LVOT) plays a major role in causing symptoms such as angina, syncope, and dyspnea. Pharmacological treatment is commonly used and includes beta-blockers, calcium channel blockers, and, more recently, mavacamten.^1-3^ However, about 10% of patients do not respond to medical therapy.^4^ For these individuals, septal reduction therapies (eg, surgical myectomy and alcohol septal ablation [ASA]) have been developed to relieve symptoms and improve clinical outcomes.^5,6^

Surgical myectomy is still considered the gold-standard treatment for hypertrophic obstructive cardiomyopathy, especially in younger patients with high baseline gradients, marked septal hypertrophy, or additional surgical needs.^7^ However, advances in ASA (eg, contrast-enhanced echocardiography and more precise alcohol delivery) have improved its outcomes. In selected patients, ASA has shown similar long-term mortality rates (around 1%) and fewer periprocedural complications.^5,6^ Myectomy is generally preferred for younger, low-risk patients with septal thickness greater than 25 mm. In contrast, ASA is often recommended for older, high-risk patients with suitable anatomy because it is less invasive.^5^

Current guidelines recognize both surgical myectomy and ASA as valid treatment options, but careful evaluation by a heart team is essential. This assessment should take into account the patient’s overall condition, the specific risks of each procedure, and the presence of mitral valve abnormalities or other anatomical considerations.^5^ Despite global experience, there is a lack of up-to-date data on the use of ASA for treating HOCM in Brazil.

The aim of this study is to evaluate the safety and effectiveness of ASA in a series of symptomatic HOCM patients on optimal medical therapy using current techniques across multiple Brazilian centers.

## Methods

### Study population

Patients who underwent ASA between January 2014 and December 2023 were retrospectively recorded in a dedicated database. Procedures were performed at four tertiary referral centers in Brazil: Instituto do Coração Hospital das Clínicas da Faculdade de Medicina da Universidade de São Paulo (InCor-HCFMUSP), Hospital Sírio-Libanês, Hospital São Camilo, and Hospital Samaritano Paulista. The study was approved by the local ethics committee at each participating center.

Patients were eligible if they were 18 years or older and had a diagnosis of HOCM confirmed by 2-dimensional echocardiography and/or cardiac magnetic resonance imaging (CMR). Diagnosis required an interventricular septum (IVS) thickness ≥15 mm, or ≥13 mm in patients with a first-degree adult relative diagnosed with HOCM in the absence of other cardiac or systemic diseases associated with secondary LV hypertrophy.

Obstruction was defined as a LVOT pressure gradient of ≥50 mmHg either at rest or after provocation. Eligible patients also had to be symptomatic and refractory to optimized medical therapy, including at least one negative chronotropic agent at the maximum tolerated dose, and present with New York Heart Association (NYHA) class ≥ II dyspnea or Canadian Cardiac Society (CCS) class ≥ II angina.^6^ During the study period, mavacamten was not yet available to patients in this cohort.

In addition, patients had to be considered suitable for septal reduction therapy based on clinical indication and favorable coronary anatomy. This included having an accessible septal branch — typically originating from the left anterior descending (LAD) artery — and no significant intrinsic abnormalities of the mitral valve or other conditions that would require cardiac surgery.^7,8^

Exclusion criteria included the absence of follow-up data beyond the index hospitalization (eg, missing clinical or echocardiographic follow-up) and unfavorable coronary anatomy for ASA, including the absence of a suitable septal branch, septal thickness >30 mm, or the need for concomitant surgical procedures.

### ASA procedure

All procedures followed the standard ASA technique, performed under contrast-enhanced echocardiography as previously described.^9^ Patients received either sedation or general anesthesia, with either contrast-enhanced transesophageal or transthoracic echocardiography guidance. After arterial and venous access were obtained, systemic anticoagulation was initiated with unfractionated heparin at 100 IU/kg.

A temporary pacemaker lead (5F or 6F) was placed in the right ventricle. A 5F or 6F pigtail catheter was positioned in the left ventricle via secondary access, and a 6F left guiding catheter (Judkins Left [JL] or Extra-Backup [EBU]) was introduced through primary access into the ascending aorta. The LVOT pressure gradient was continuously monitored throughout the procedure.

Initial angiography was performed to identify the origin of the septal perforator branch. A 0.014” guidewire was then advanced into the target septal branch. Afterward, an over-the-wire balloon (1.5-2.5 mm × 8-12 mm) was inflated within the selected branch. Contrast-enhanced echocardiography was used to assess myocardial perfusion of the target area. In 58.5% of cases, SonoVue (Bracco) was used; in the remaining cases, regular saline mixed with contrast was injected through the balloon catheter shaft during real-time echocardiographic recording.

If the balloon was correctly positioned, 1 to 3 mL of ethanol was slowly injected at a rate of 1 mL per minute. Ten minutes after the injection, the balloon was deflated, and coronary angiography was repeated to assess the result. A successful procedure was defined as a reduction of at least 50% in the LVOT pressure gradient compared to baseline. If the outcome was unsatisfactory, the procedure could be repeated in a different septal branch.

All patients were monitored in an intensive care or coronary care unit for 24 to 48 hours after the procedure. Plasma creatine phosphokinase (CPK) and creatine kinase-MB (CK-MB) levels were measured before the procedure and every 6 hours for 36 hours afterward.

Echocardiographic evaluations were performed before ASA, and again at 1 month and 1 year after the procedure. All measurements followed the recommendations proposed by the American Society of Echocardiography.^10-12^

### Definitions and study endpoints

The primary efficacy endpoint was defined as a reduction of >50% in the resting maximum LVOT gradient, with a final gradient <50 mmHg. This was used to determine procedural success and was assessed at 1 and 12 months after ASA. Based on this criterion, patients were classified as responders or nonresponders.

Improvement in cardiac symptoms was evaluated by a reduction of at least one functional class in either the NYHA or CCS classification following the procedure.

The primary safety endpoint was the occurrence of complications, including complete atrioventricular block, ventricular fibrillation, implantation of a permanent pacemaker or cardioverter defibrillator, cardiac tamponade, emergency cardiac surgery, or cardiovascular death.

### Statistical analysis

Statistical analyses were performed using GraphPad version 10.2.2 and Microsoft Excel 2010 (Microsoft Corporation, Redmond, Washington). Categorical variables are presented as absolute and relative frequencies.

Normality was assessed using the Shapiro-Wilk test. Data with normal distribution are reported as mean±standard deviation, while non-normally distributed data are expressed as median and interquartile range (IQR).

To compare continuous variables between two independent groups, the unpaired Student’s t-test was used for normally distributed data, and the Mann-Whitney U test was applied for non-normally distributed data. For comparisons involving more than two time points, repeated-measures analysis of variance (ANOVA) was used when data were normally distributed.

Categorical variables in independent samples were compared using the Chi-square test. For dependent categorical data, Cochran’s Q test was applied.

Kaplan-Meier survival curves were analyzed using the log-rank test. In all analyses, a p-value <0.05 was considered statistically significant.

## Results

### Baseline characteristics and ASA procedures

A total of 46 patients were included in the registry. However, five were excluded due to missing follow-up data — two lacked clinical information, and three had no echocardiographic data (Figure 1). The mean age was 66 years, and most patients were female (73%). The main indication for ASA was exertional dyspnea. Most patients were classified as NYHA class III/IV (78.1%) or CCS class III/IV (51.1%). Additionally, 24.3% had a history of syncope not related to arrhythmias. In total, 93.2% of patients presented with advanced symptoms.

**Figure 1:** – Flowchart of the studied population.

Common comorbidities included hypertension, diabetes mellitus, atrial fibrillation, and coronary artery disease. Some patients had undergone prior coronary angioplasty or presented with conduction abnormalities, such as left bundle branch block. A small number had implantable cardioverter-defibrillators (ICDs). All patients were on beta-blockers or calcium channel blockers at baseline, with beta-blockers being more frequently used. A smaller proportion required amiodarone or oral anticoagulants. CMR was used to assess myocardial fibrosis. The average fibrosis area was 11.7%, with no significant differences between groups (Table 1).

**Table 1:** – Baseline clinical and echocardiographic/CMR characteristics

Variable	Baseline (n=41)	Responders (n=30)	Nonresponders (n=11)	p-value
Age (years)	66.4±5.9	64.7±6.8	65.4±7.2	0.64
Female sex, n (%)	30 (73.1)	24 (80.0)	6 (54.5)	0.81
Hypertension, n (%)	27 (65.8)	21 (70.0)	6 (54.5)	0.74
Coronary artery disease, n (%)	7 (17.0)	3 (10.0)	4 (36.3)	0.67
Previous PCI, n (%)	3 (7.2)	2 (6.6)	1 (9.0)	0.36
Diabetes mellitus, n (%)	9 (21.0)	7 (23.3)	2 (18.1)	0.68
NYHA dyspnea class III/IV, n (%)	32 (78.0)	25 (83.3)	7 (63.6)	0.68
CCS angina III/IV, n (%)	21 (51.2)	15 (50.0)	6 (54.5)	0.75
Atrial fibrillation, n (%)	4 (9.7)	3 (10.0)	1 (9.0)	0.75
Bundle branch block, n (%)	6 (14.6)	4 (13.3)	2 (18.1)	0.45
LVOT gradient at rest (mmHg)	88.4±23.0	73.4±23.4	112.6±40.2	0.04
LVEF (%)	66.4±11.1	65.3±9.1	68.2±10.4	0.92
IVS thickness (mm)	19.3±3.2	15.3±2.2	16.5±3.9	0.56
Beta‐blocker, n (%)	35 (85.3)	25 (83.3)	10 (90.9)	0.33
Calcium‐channel blocker, n (%)	10 (24.3)	8 (26.6)	2 (18.1)	0.41
Amiodarone, n (%)	6 (14.6)	4 (13.3)	2 (18.1)	0.55
Oral anticoagulant, n (%)	3 (7.3)	3 (13.3)	1 (9.0)	0.62
Fibrosis by CMR (total area, %)	11.7±4.2	10.4±3.7	12.1±2.2	0.48
Fibrosis by CMR (grams)	13.9±3.8	13.4±4.1	15.8±4.1	0.37

Data are expressed as mean (SD) or as absolute number and percentage as appropriate. CCS: Canadian Cardiovascular Society; CMR: cardiac magnetic resonance imaging; ICD: implantable cardioverter-defibrillator; IVS: interventricular septum; LVEF: left ventricular ejection fraction; LVOT: left ventricular outflow tract; NYHA: New York Heart Association; PCI: percutaneous coronary intervention; SD: standard deviation.

Baseline Doppler echocardiography confirmed the presence of severe HOCM, characterized by a high LVOT pressure gradient and increased IVS thickness. Most patients (92.6%) had preserved LV ejection fraction (LVEF), and the left atrium was moderately enlarged (Table 1). The median length of hospital stay was 6 days, with a range of 3 to 15 days. Laboratory tests showed stable values for hemoglobin, hematocrit, and creatinine throughout hospitalization. Refractoriness to pharmacological treatment was observed in 18.2% of patients. Such individuals were on combined therapy with beta-blockers and calcium channel blockers but continued to show elevated LVOT gradients despite optimal medical management (Table 1).

The ASA procedure was performed using a median ethanol volume of 1.9 cc per patient. Most patients (n=36) underwent ASA of a single septal branch, while five required the treatment of two septal segments during the same procedure. Post-procedural levels of peak CK-MB and troponin were elevated but remained within the expected range (Table 2). The radial artery was used as the preferred access site in 73.1% of cases, and most procedures were performed with a 6F catheter (Table 2).

**Table 2:** – Alcohol septal ablation procedural characteristics

Procedural characteristics	Baseline (n=41)	Responders (n=30)	Nonresponders (n=11)	p-value
Mean number of septal branches treated per patient	1.12±0.07	1.13±0.09	1.09±0.04	0.83
One septal branch treated, n (%)	36 (87.8)	26 (86.6)	10 (90.9)	
Two septal branches treated, n (%)	5 (12.2)	4 (13.4)	1 (9.1)	
Ethanol volume per patient (mL), median [IQR]	1.9 [1.6-2.4]	1.8 [1.6-2.3]	2.0 [1.8-2.4]	0.54
Peak CK-MB (ng/mL), median [IQR]	157 [126-254]	152 [113-286]	169 [129-304]	0.42
Peak troponin (ng/L), median [IQR]	10.860 [9.576-12.239]	10.539 [9.284-12.482]	11.285 [9.896-12.129]	0.39
Primary arterial access, n (%)				0.71
Radial	30 (73.1)	23 (76.6)	7 (63.6)	
Femoral	11 (26.8)	7 (23.4)	4 (36.4)	
Catheter diameter, n (%)				0.63
6F	38 (92.7)	28 (93.3)	10 (90.9)	
5F	3 (7.3)	2 (6.7)	1 (9.1)	

Data are expressed as absolute number and percentage, mean (SD) or median [IQR], as appropriate. CK-MB: creatine kinase-MB; IQR: interquartile range; SD: standard deviation.

### Efficacy outcomes

Following the ASA procedure, there was a significant reduction in the number of patients classified as NYHA class III/IV. At 1 month, 26.8% remained in these advanced classes, and this dropped further to 17.0% at 12 months. A similar improvement was seen in CCS classification, with 12.1% and 9.7% of patients remaining in class III/IV at 1 month and 12 months, respectively (Figure 2). Echocardiographic assessments showed a substantial reduction in the LVOT pressure gradient, decreasing from 88.4 mmHg to 27.0 mmHg. IVS thickness also decreased, from 19.3 mm to 14.7 mm at 12 months after the procedure (Figure 3).

**Figure 2:** – Clinical follow-up. CCS: Cardiovascular Canadian Society; NYHA: New York Heart Association; Pre: prior to the procedure.

**Figure 3:** – Echocardiographic changes over time. CCS: Cardiovascular Canadian Society; NYHA: New York Heart Association; SAM: systolic anterior motion; IVS: interventricular septum.

Systolic anterior motion (SAM) of the mitral valve, which reflects the Venturi effect caused by LVOT obstruction, also showed a significant reduction—from 51.2% before the procedure to 19.5% at 12 months. Notably, the incidence of SAM continued to decline between the 1^st^ and 12^th^ month after ASA, dropping from 34.1% to 19.5% (Figure 3).

Based on a reduction of at least 50% in the LVOT pressure gradient and a final absolute value <50 mmHg, patients were classified into two groups: responders (30 patients, 73.0%) and nonresponders (11 patients, 27.0%). The responder group had a lower baseline LVOT pressure gradient compared to the non-responder group (73.4±23.4 mmHg vs 112.6±40.2 mmHg) (Table 1 and Figure 4). However, there was no significant difference in baseline IVS thickness between the two groups, as measured by echocardiography (15.3±2.2 mm vs 16.5±3.9 mm) (Table 1 and Figure 4). A receiver operating characteristic (ROC) curve analysis identified a baseline LVOT gradient of 105 mmHg as the optimal cutoff to predict response to ASA (Figure 4). During a median follow-up of 394 days, Kaplan-Meier analysis showed that nonresponders had significantly higher rates of hospitalization compared to responders (Figure 5).

**Figure 4:** – Comparison between responders vs nonresponders (baseline echocardiographic parameters). ASA: alcohol septal ablation; IVS: interventricular septum.

**Figure 5:** – Comparison between responders vs nonresponders hospitalization after alcohol septal ablation (ASA) procedure.

Two patients from the nonresponder group underwent surgical myectomy after ASA. Both had residual LVOT gradients >50 mmHg. Following surgery, the gradient was no longer present on echocardiographic evaluation, and no complications were reported during the procedure or follow-up.

### Safety outcomes

Complete atrioventricular block occurred peri-procedurally in seven patients (17%). Among these, two patients (4.8%) returned to sinus rhythm within 24 hours, and three patients (7.3%) did so within 48 hours. Permanent pacemaker implantation was required in two patients (4.8%). During the study period, a five-day observation window was maintained before deciding on pacemaker implantation. Ventricular fibrillation occurred during the procedure in four patients (9.7%) but was successfully reversed in all cases, with favorable postoperative recovery. There were no cases of cardiac tamponade, emergent cardiac surgery, or in-hospital mortality. No deaths were reported during the median mid-term follow-up of 394 days (Table 3).

**Table 3:** – Safety outcomes of the alcohol septal ablation procedure

Safety outcomes	Overall (n=41)	Responders (n=30)	Nonresponders (n=11)
Complete atrioventricular block, n (%)	7 (17)	5 (16.6)	2 (18.1)
New pacemaker implantation, n (%)	2 (4.8)	1 (3.3)	1 (9)
Ventricular fibrillation, n (%)	4 (9.7)	3 (10)	1 (9)
Cardiac tamponade	—	—	—
Urgent heart surgery	—	—	—
Mortality	—	—	—

Data are expressed as absolute number and percentage

## Discussion

The main findings of this study are as follows: i) there was significant clinical improvement after the ASA procedure, with fewer than 20% of patients remaining in CCS class III/IV or NYHA class III/IV at 12 months; ii) echocardiographic evaluation showed a clear reduction in the LVOT pressure gradient, along with a decrease in IVS thickness and a marked reduction in SAM of the mitral valve; iii) 73% of patients showed a positive response to ASA, which was associated with fewer hospitalizations during follow-up, with a baseline intraventricular gradient <105 mmHg being the best predictor of response; iv) ASA proved to be a safe procedure in this cohort, with a low complication rate and no reported mortality.

HOCM is a heterogeneous condition with high prevalence in our population. Although most patients remain stable with medical therapy, around 10% may eventually require septal reduction procedures such as surgical myectomy or ASA. Over the past decades, the ASA technique has evolved and incorporated improvements such as contrast-enhanced echocardiography and lower volumes of alcohol injection. However, there is still a lack of data evaluating consecutive patients treated with ASA in our region.^11-13^ Our cohort, though limited to 41 patients, included individuals with advanced HOCM, characterized by a high symptom burden — 78% were in NYHA class III/IV and 51% in CCS class III/IV — and a mean fibrosis extent of 11.7%. The small sample size likely reflects a low referral rate for septal reduction therapies, whether surgical myectomy or ASA, in patients with HOCM.

During ASA, the acute reduction in the LVOT gradient is likely due to septal stunning or infarction^2-5^ and changes in LV ejection dynamics.^14^ These factors may contribute to short-term symptom relief. As reported in literature, progressive septal thinning and left ventricular remodeling are the main drivers of the longer-term reduction in gradient observed at 12 months.^14^ This sustained improvement is further supported by the continued reduction in symptoms and the decline in LVOT gradient over time.^15^

No significant association was found between septal thickness and procedural success. This contrasts with previous studies suggesting that IVS thickness >30 mm may reduce the effectiveness of ASA, making myectomy the preferred approach in such cases. However, surgical outcomes in patients with very thick septa are also often suboptimal.^13,16^ Notably, our study did not include any patients with IVS thickness >30 mm. This may reflect a selection bias since such patients were likely referred directly for surgical intervention.

A successful ASA procedure — defined as a reduction of more than 50% in the LVOT gradient and a final absolute value <50 mmHg — was achieved in 73% of our patients (n=30), consistent with findings from previous studies.^17-19^ As also reported in literature,^2,20^ patients who responded to ASA showed clinical improvement, with about 50% reaching NYHA class I.^21,22^ Most of these responders had baseline LVOT gradients <100 mmHg prior to the procedure.

Recent data from a cohort of 1,346 patients who underwent ASA followed for an average of 5.8±4.7 years showed that individuals with a baseline LVOT gradient ≥100 mmHg had higher residual gradients and were more likely to require repeat ASA or additional myectomy, compared to those with baseline gradients <100 mmHg.^14^ In our study, a negative correlation was observed between baseline LVOT gradient and ASA outcomes. We identified a potential cutoff of 105 mmHg as a predictor of procedural success. This finding highlights the importance of assessing baseline LVOT gradient when selecting candidates for ASA, although confirmation in larger studies is still needed.

In the present study, ASA was performed using contemporary techniques by experienced teams at tertiary centers.^11,16,23,24^ The use of transthoracic echocardiography combined with contrast injections improved the accuracy of the procedure. Additionally, the use of intracoronary balloons for selective alcohol injection into the target septal branch increased procedural safety. These technical advancements contributed to the low complication rate observed in our cohort. Permanent pacemaker implantation was required in only 4.8% of patients, representing a significant reduction compared to rates reported in earlier studies. Temporary pacemakers were used in 17% of cases, emphasizing the need for close monitoring during the first 48 hours to assess whether permanent implantation is necessary. Recovery of sinus rhythm during this observation period may depend on several factors, including the extent of myocardial damage, patient characteristics, and procedural technique.^24^

This study has some limitations. First, it was a retrospective analysis with a small sample size of 41 patients who underwent a single procedure. As a result, selection bias may be present. Second, the absence of post-procedure stress testing introduces a potential limitation in the assessment of symptom improvement. No standardized questionnaire was used; instead, symptom data were based on NYHA and CCS classifications, relying on patients’ subjective reports during routine clinical visits. Additionally, syncope as a primary indication for ASA was not recorded in our database, so its prevalence in this cohort could not be assessed. Finally, there was a loss of clinical and echocardiographic follow-up data after 12 months, which limited the evaluation of long-term outcomes.

## Conclusion

When performed with proper indications and advanced techniques (eg, contrast-enhanced echocardiography) ASA is a safe, effective procedure with a low complication rate. It results in significant improvements in echocardiographic parameters, including reductions in LVOT gradient and IVS thickness, leading to symptom relief. This study identified a baseline LVOT gradient <105 mmHg as a potential predictor of favorable ASA outcomes. These findings underscore the critical importance of meticulous patient selection and the use of contemporary ASA techniques to optimize clinical results.

### Clinical perspectives

The BRASA registry highlights the growing role of ASA as a safe, effective treatment for patients with symptomatic HOCM who do not respond to medical therapy. The study shows ASA leads to symptom relief and echocardiographic improvements, including reductions in LVOT gradient and IVS thickness, reinforcing its potential to improve quality of life in selected patients.

A baseline LVOT gradient <105 mmHg was identified as a possible predictor of favorable outcomes, supporting its use in guiding patient selection. The study also emphasizes the value of contemporary ASA techniques—such as contrast-enhanced echocardiography and precise alcohol delivery—which contribute to improved safety and efficacy. Low complication rates, particularly reduced pacemaker implantation, underscore the importance of standardized techniques and experienced operators. Further research is needed to confirm these findings, better define the role of ASA compared to emerging therapies such as mavacamten, and clarify its position in the HOCM treatment pathway.
